# Case report: First manifestation of multiple sclerosis temporally correlated with COVID-19 vaccination

**DOI:** 10.3389/fneur.2023.1097799

**Published:** 2023-02-15

**Authors:** Agata Czarnowska, Katarzyna Kapica-Topczewska, Eugeniusz Tarasów, Joanna Tarasiuk, Monika Chorąży, Jan Kochanowicz, Alina Kułakowska

**Affiliations:** ^1^Department of Neurology, Medical University of Białystok, Białystok, Poland; ^2^Department of Radiology, Medical University of Białystok, Białystok, Poland

**Keywords:** multiple sclerosis, autoimmune condition, vaccination, SARS-CoV-2, COVID-19, case report

## Abstract

There are several case reports describing a temporal correlation between the first clinical manifestation of multiple sclerosis (MS) and the occurrence of relapses with vaccination against SARS-CoV-2. Here we report a case of a 33-year-old male who developed partial right upper and lower extremities numbness 2 weeks after receiving Johnson & Johnson's Janssen COVID-19 vaccine. The brain MRI performed during diagnostics in the Department of Neurology detected several demyelinating lesions, one with enhancement. Oligoclonal bands were present in the cerebrospinal fluid. The patient was treated with high-dose glucocorticoid therapy with improvement and the diagnosis of MS was made. It seems plausible that the vaccination revealed the underlying autoimmune condition. Cases like the one we reported here are rare, and—based on current knowledge—the benefits of vaccination against SARS-CoV-2 far outweigh the potential risks.

## Introduction

Among many environmental factors contributing to the pathogenesis of multiple sclerosis (MS), it is suspected that infectious agents play a triggering role. The most often implicated are the Herpesviridae family viruses, the John Cunningham virus, and human endogenous retroviruses (1). There are several case reports describing a temporal correlation between the first clinical manifestation of MS and the occurrence of relapses with vaccination against SARS-CoV-2 (2, 3). The data are still very limited, and the cause-and-effect relationship is vague, but those cases need to be noted. Furthermore, the COVID-19 pandemic is ongoing, and such data will be crucial for future analysis.

## Case report

**Figure 1:**
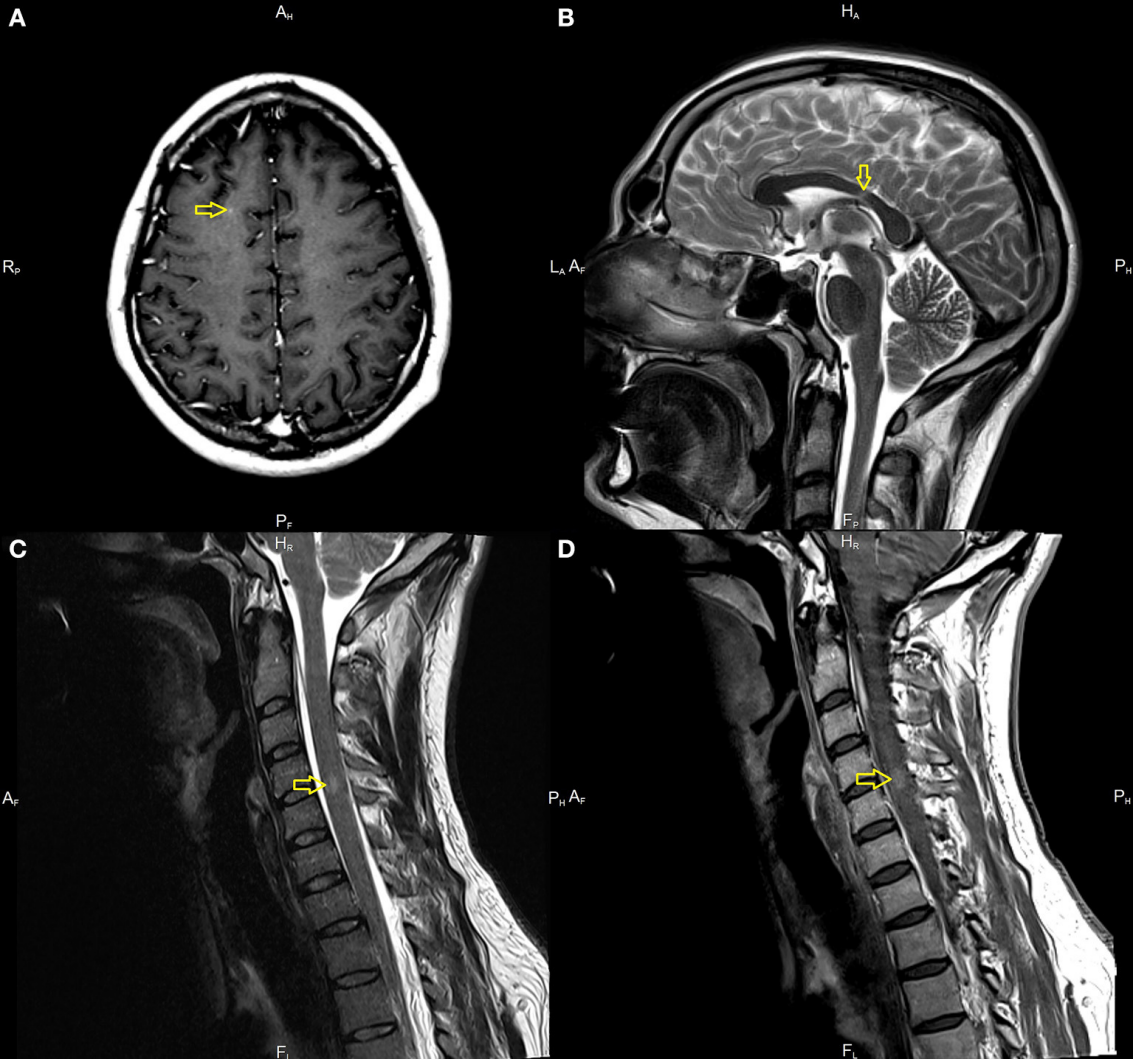
Magnetic resonance imaging (MRI) in the described case of multiple sclerosis (MS). Demyelination lesions are marked with yellow arrows, respectively. **(A)** T1-weighted subcortical Lesion with gadolinium enhancement on brain MRI on axial sequences. **(B)** T2-weighted images showing lesion located in corpus callosum. **(C)** T2 hyperintense acute lesion on cervical spinal cord on sagittal sequences. **(D)** Gadolinium enhancement of a lesion located in the cervical spinal cord.

A 33-year-old male with a history of mild, well-controlled hypertension developed right upper and lower extremities numbness 2 weeks after receiving Johnson & Johnson's Janssen COVID-19 vaccine. In the prior period, the patient was not positive for coronavirus disease 2019 (COVID-19) infection and did not present any symptoms suggesting the presence of SARS-CoV-2 infection. Due to the above symptoms, the patient was admitted to the Department of Neurology. The examination revealed right side sensory impartment (loss of pain and temperature feeling) at the C5 level. Magnetic resonance imaging (MRI) scans of the brain and thoracic and cervical spine were performed. The brain MRI detected several demyelinating lesions, one with enhancement (in the subcortical region of the right frontal lobe). The spinal cord imaging showed an enhancing lesion consistent with demyelination at the fourth/fifth cervical vertebrae (C 4/5) and one smaller lesion at the level of the third/fourth cervical vertebrae (C3), without enhancement (Figure 1). Cerebrospinal fluid (CSF) analysis demonstrated moderate pleocytosis (25 leukocytes/μl; normal range 0–5 leukocytes/μl), elevated IgG index (0.84; normal range 0.0–0.7), and the presence of oligoclonal bands (serum unmatched CSF oligoclonal bands). Other evaluations from serum and CSF were unremarkable (serum: C-reactive protein, erythrocyte sedimentation rate, blood count were in the limits of the laboratory standard; CSF: glucose value 76 mg/dl with normal range of 50–80 mg/dl, protein value 44 mg/dl with normal range of 15–45 mg/dl). Diagnostic tests for infectious were negative. These included an examination of the presence of *Borrelia burgdorferi* and tick-borne encephalitis virus; the region of the patient living is endemic for both. Tests for the presence of anti-aquaporin-4 antibodies, myelin basic protein antibodies, and antibodies to myelin oligodendrocyte glycoprotein were negative. The evaluation for other autoimmune diseases was negative and included: rheumatoid factor, antiprothrombin, antinuclear, antiphospholipid, and antineutrophil cytoplasmic antibodies, antigens against :dsDNA, nucleosome, histosome, SS-A, Ro-52, SS-B, nRNP/Sm, Sm, Mi-2alfa, Mi-2beta, Ku, A, and B centromeres, Sp100, PML, PM-Scl100, PM-Scl75, Scl-70, RP155, gp210, PCNA, and DFS70.

The patient was treated with high-dose glucocorticoid therapy (1,000 mg of methylprednisolone i.v. for 3 days). A significant improvement in the patient's neurological condition was achieved but without complete recovery. A subtle disturbance of temperature sensation persisted (discharge EDSS = 1.0). The patient was diagnosed with MS according to the 2017 McDonald criteria and started treatment with dimethyl fumarate (4). In 1 year of observation, no relapses were present, there was no worsening of the patient's neurological state (EDSS = 1.0), and initial treatment with disease-modifying therapy has been continued. The follow-up MRI showed no new lesions and no enhancing lesions.

## Discussion

In most countries, sanitary restrictions due to the spread of SARS-CoV-2 are now limited. However, the circulation of new variants of the virus is ongoing. Therefore, broad immunization is crucial for the further preservation of global health.

Vaccination against SARS-CoV-2 is recommended for individuals with MS (5). The serological response to SARS-CoV-2 vaccination is satisfactory in most patients treated with disease-modifying therapies (DMTs). Lower seroconversion is noted in individuals treated with anti-CD20 therapies and fingolimod (6).

In one of our previous works, we collected a database of more than 2000 individuals with MS from Poland, and the relapse rate after vaccination against COVID-19 was low (7). Similarly, low relapse incidence after vaccination was described by Kong et al. (8), including individuals with neuromyelitis optica spectrum disorder (NMOSD). In the analysis of patients with MS from Germany and the United Kingdom, the percentage of individuals manifesting deterioration after vaccination was significantly higher (19%), but not only relapses were taken into account (9). In the study of Stastna et al. (10), the relapse incidence was slightly higher in vaccinated individuals, but also in people with SM who had a history of COVID-19 infection. An interesting observation was made by Brunn et al. (11), where among about 300 vaccinated people with MS, worsening was mostly associated with symptom recrudescence (not new deficits). There are also case series showing the time relation of AstraZeneca vaccination to relapse in previously stable patients (12). However, studies with more people presenting worsening following immunization do not show the predominance of a particular vaccine (13). Based on currently available data, the potential for available vaccinations to induce autoimmune exacerbation, in general, is not significantly high. What is more, most authors agree, that the benefit of vaccination, for now, outweighs the potential risk of triggering relapse (14).

The presented case and similar cases reported by other authors are rare. The only link between the onset of symptoms and immunization is the time interval. It is impossible to state unequivocally that the vaccination triggered the onset of symptoms or that this was coincidental.

In the presented case, it seems plausible that the vaccination revealed the underlying autoimmune condition. The MRI scans showed dissemination in time. This indicates the presence of a latent autoimmune process before immunization. A case of exacerbation after vaccination with the Johnson & Johnson COVID-19 vaccine in an individual with MS, previously presenting neurological symptoms, is described in the literature (15). In the case we are presenting, the patient has never had any neurological symptoms before immunization. Other similar case reports (with new onset of the disease) in the literature followed the Pfizer-BioNTech COVID-19, Moderna COVID-19, and Oxford Astra Zeneca COVID-19 vaccines (3, 16, 17). There are reports of other CNS inflammatory demyelinating events (acute transverse myelitis, NMOSD, myelin oligodendrocyte glycoprotein antibody disease, acute disseminated encephalomyelitis) following vaccination against COVID-19 (18).

How the COVID-19 pandemic will unfold is difficult to predict. However, the current experiences have shown that broad immunization with currently available vaccines is safe and effective, reducing the risk of severe COVID-19, hospitalization, and death (19). Multiple studies have concluded that COVID-19 vaccines are safe for individuals with MS (20–22). Cases like the one we reported here are rare, and—based on current knowledge—the benefits of vaccination against SARS-CoV-2 far outweigh the potential risks.

## Data availability statement

The datasets presented in this article are not readily available because of ethical and privacy restrictions. Requests to access the datasets should be directed to the corresponding author.

## Ethics statement

The studies involving human participants were reviewed and approved by Bioethics Committee at Medical University of Białystok. The patients/participants provided their written informed consent to participate in this study. Written informed consent was obtained from the individual(s) for the publication of any potentially identifiable images or data included in this article.

## Author contributions

AC, KK-T, JT, MC, JK, and AK contributed to conception and design of the article. ET performed analysis of the neuroimaging. AC wrote the first draft of the manuscript. KK-T, JT, and MC revised the final version of the manuscript. All authors contributed to manuscript revision, read, and approved the submitted version.
